# Association of Sleep Quality and Waking Time with Prediabetes: The Qazvin Metabolic Diseases Study, Iran

**DOI:** 10.1155/2015/480742

**Published:** 2015-08-16

**Authors:** Azam Ghorbani, Neda Esmailzadehha, Asghar Mohammadpoorasl, Amir Ziaee

**Affiliations:** ^1^Metabolic Diseases Research Center, Qazvin University of Medical Sciences, Qazvin 3413786165, Iran; ^2^Tabriz Health Services Management Research Center, Tabriz University of Medical Sciences, Tabriz, Iran

## Abstract

*Aims*. It is known that sleep has a major role in the regulation of endocrine functions and glucose metabolism. However, it is not clear whether the sleep pattern is affected at or prior to the onset of diabetes, among those with prediabetes. The purpose of this study was to determine the association of sleep patterns and prediabetes in Qazvin, Iran. *Methods*. A representative sample of residents of Qazvin was selected by multistage cluster random sampling method in 2011. Plasma glucose level and sleep quality were measured cross-sectionally as well as demographic characteristics. A logistic regression analysis was used to examine the association of sleep status and prediabetes. *Results*. Mean age was 39.3 ± 10.1 years. Of 958, 474 (49.47%) were female. Poor sleep quality was associated with 2.197-fold increased risk of prediabetes after adjustment for age, gender, body mass index, and metabolic syndrome. *Conclusion*. This study provides evidences that subjects with poor sleep quality are more likely to develop prediabetes than people with good sleep quality.

## 1. Introduction

Sleep is one of the important elements in human life which is associated with reconstruction of physical and emotional power. Maintaining regular sleep cycles is absolutely necessary in order to preserve fitness and health. Modern society encourages late night activities, such as watching television, using the computer or Internet, and round-the-clock entertainment, as well as demanding shift work or night work that further promotes such activities [1].

From four decades ago, several studies showed that sleep has a major role in the regulation of endocrine functions and glucose metabolism [2, 3]. Sleep fragmentation leads to an increase in sympathetic nervous system activity [4] that inhibits insulin secretion and promotes insulin resistance [5]. Short sleep duration leads to changes in levels of appetite-regulating hormones [6] and increases energy intakes without increase in energy expenditure that make people at risk for obesity [7].

Glucose tolerance and insulin secretion are also markedly modulated by the sleep waking cycle [8]. Rafalson et al. after 6 years of follow-up showed that short sleep duration was associated with threefold increased risk of developing impaired fasting glucose (IFG) even after considering several putative risk factors of diabetes [9]. Some prospective studies have reported that extreme sleep duration [10, 11] and poor sleep quality, such as difficulty in sleep initiation [12], are associated with a higher risk of impaired glucose tolerance or developing type 2 diabetes.

However, it is not clear whether the sleep pattern is affected at or prior to the onset of diabetes, among those with prediabetes. Therefore, the authors hypothesized that poor sleep quality and short sleep duration are associated with increased risk of prediabetes. The present study was designed to examine this hypothesis and determine the association of sleep patterns and prediabetes in Qazvin, Iran.

## 2. Material and Methods

This cross-sectional study was performed on a representative sample of residents of Minoodar district of Qazvin, which is located 150 km northwest of Tehran capital of Iran, from September 2010 to April 2011. The study was approved by the ethics committee of Qazvin University of Medical Sciences.

The sampling unit was household and all households had health profiles at the health center located in the district. The Minoodar district was divided into four main clusters according to the population size. The households were selected by multistage cluster random sampling methods. The inclusion criterion was age ≥ 20 yr. Subjects were invited by telephone to attend the study and after explanation of the complete details, they were free to participate. All subjects gave their written informed consent. Finally, 1107 people were selected for the study. Demographic and social data were self-reported in the questionnaire given to the subjects. Two general practitioners filled out an organized questionnaire including medical history and physical examination. Details of sampling method and data collection have been published elsewhere [13, 14].

Plasma glucose level was measured after a 12–14 h overnight fasting. An oral glucose tolerance test (OGTT) was performed for all subjects without known diabetes by 75 g glucose. According to the American Diabetes Association classification [15], impaired fasting glucose (IFG) was defined as fasting plasma glucose (FPG) levels ≥5.6 mmol/L but <7.0 mmol/L; impaired glucose tolerance (IGT) was defined as 2-h values in the OGTT test ≥7.8 mmol/L but <11.1 mmol/L; diabetes was defined as fasting plasma glucose ≥7.0 mmol/L or 2-h postload glucose ≥11.1 mmol/L during an OGTT test, or previously diagnosed diabetes. IFG and IGT were considered as prediabetes. In the present study, subjects with diabetes were excluded. The study population was divided in two groups including subjects with normal glucose metabolism and those with prediabetes. Metabolic syndrome was defined according to criteria proposed by national cholesterol education program third adult treatment panel [16].

Sleep quality was assessed by the Pittsburgh sleep quality index (PSQI) which evaluates sleep quality over a 1-month time interval [17]. The PSQI is a 19-item self-rated questionnaire that generates seven sleep component scores on a 0–3 scale, with three indicating the greatest dysfunction. A global PSQI score is composed of the sum of scores for the seven components in a way that higher score indicates worse sleep quality. Poor sleep quality was defined as PSQI score greater than 5 [17].

Self-reported sleep duration was assessed with questions about bedtime and waking time for each subject. Sleep duration was classified into three groups: less than 6 hours, between 6 and 8 hours, and more than 8 hours. Waking time was classified into three groups: earlier than 6.00 am; 6-7 am; and after 7.00 am. Late sleep onset was defined as bedtime after 12.00 am.

Data were recorded as mean ± standard deviation (SD) or number (percent). The PSQI factors were compared between subjects with prediabetes and subjects with normal glucose metabolism using Mann-Whitney *U* test. Categorical variables were analyzed using chi-square test. A logistic regression analysis was used to examine the association of sleep status and prediabetes. *P* values less than 0.05 were considered as statistically significant. All of the analyses were performed using the SPSS software, version 22.

## 3. Results

A total of 982 participants (20–72 years old) enrolled in the study. Of these, 958 had complete questionnaires and laboratory tests. Mean age was 39.3 ± 10.1 years. Of 958, 474 (49.47%) were female, and 27% had prediabetes. Subjects with prediabetes were older than normal subjects (43.8 ± 9.5 versus 37.6 ± 9.8; *P* < 0.001). Prediabetes was more prevalent in males than in females (32.1% versus 22.1%; *P* = 0.001).

The total global PSQI score was 8.37 ± 2.7. This score was 8.27 ± 2.7 in the subjects with normal glucose metabolism and 8.35 ± 2.5 in the subjects with prediabetes. The difference was borderline significant between two groups (*P* = 0.053). PSQI scores of the study subjects are shown in Table 1. Only sleep duration and sleep disturbances scores were significantly higher in the subjects with prediabetes compared to the subjects with normal glucose metabolism.

The relationship between sleep patterns and glucose metabolism status is shown in Table 2. In univariate analysis, waking time was associated with prediabetes while sleep duration and bedtime were not associated with prediabetes.

In logistic regression analysis, poor sleep quality was associated with 2.197-fold increased risk of prediabetes after adjustment for age, gender, body mass index, and metabolic syndrome (Table 3).

## 4. Discussion

Living in the 21st century and changes in activity patterns have a significant impact on individual's healthy sleeping habits [18]. Human behavior and sleep habits may affect internal circadian clock and homeostatic mechanism and result in alteration of sleep quality and duration [19]. Quantity and quality of sleep have important roles in regulation of glucose metabolism [20]. However, much of the evidence provided that people with diabetes have poor sleep compared to those who did not have diabetes [21] and people with prediabetes are at increased risk of diabetes [22]. In Chaput et al. study, short and long time sleep were associated with a higher risk of developing IGT and type 2 diabetes [23]. This study is one of the few to investigate the association of prediabetes with quantity and quality of sleep using an OGTT.

In the present study, subjects with prediabetes had a higher global PSQI score than those with normal glucose metabolism with borderline significance. Poorer sleep quality was also associated with 2.197-fold greater risk of prediabetes after controlling the effects of body mass index and metabolic syndrome. In support of our findings, other studies have confirmed that sleep quality is associated with incident risk for type 2 diabetes [20, 24]. Hung et al. [25] reported that subjects with newly diagnosed diabetes and prediabetes had significantly higher global PSQI scores compared to those with normal glucose metabolism. Engeda et al. found that waking during the night (≥5 times/month) was associated with 3.5 times increased risk of clinically identified prediabetes but not undiagnosed prediabetes [26]. Knutson et al. also reported that poor sleep quality and higher sleep fragmentation were associated with higher markers of glucose metabolism among subjects with diabetes [27]. These results support the notion that poor sleep quality may be a potential predictor of disordered glucose metabolism and prediabetes.

In the present study, univariate analysis showed that waking earlier than 7.00 am was associated with worse glucose metabolism compared to waking later than 7.00 am. However, this association was not confirmed in multivariate analysis. Engeda et al. in a cross-sectional study among 2285 participants from the National Health and Nutrition Examination Survey found that waking up too early more than 5 times per month was associated with clinically identified prediabetes but not undiagnosed prediabetes [26].

In the present study, short sleep duration and bedtime are not associated with prediabetes. The sample size may probably preclude finding an association between short sleep and prediabetes in this study. Chao et al. in a Taiwanese population have reported that short and long sleep durations are independent risk factors of newly diagnosed diabetes, but not prediabetes [28]. In Western New York Health Study, Rafalson et al. found that sleep duration less than 6 hours was associated with three times increased risk of developing IFG compared to midrange (6 to 8 hours) sleep duration [9]. In Engeda et al. study, short sleep (≤5 h/night) was associated with two times increased risk of clinically identified prediabetes [26]. Hayashino et al. in HIPOP-OHP study showed that the association of sleep duration and risk of diabetes was not significant [24]. In contrast, other researchers have found that short sleep duration is associated with increased risk of diabetes in Swedish and U.S. population [10, 11].

Changes due to sleep disturbances such as low amounts of slow-wave sleep may adversely affect glucose tolerance [29]. Many laboratory studies suggest multiple pathway links between sleep disturbances, either quantity or quality of sleep, insulin resistance, and glucose metabolism [19]. These evidences have revealed that insufficient sleep and sleep fragmentation alter physiological mechanisms such as diminished brain glucose utilization [30, 31]; increased sympathetic nervous system activity; and inhibited insulin secretion and promoted insulin resistance [4]. On the other hand, there are evidences that increased hunger hormone (ghrelin) levels, decreased leptin levels [32], and increased systemic inflammatory response are linked to insulin resistance [33] and are suggested underlying pathophysiology in the developments of prediabetes.

Living in modern societies and different work/social schedules often lead to mismatch in timing and circadian misalignment [19]. Scheer et al. studied 10 healthy adults under experimentally induced circadian misalignment and found that eating and sleeping 12 h after habitual times were associated with a 6% increase in plasma glucose levels [34]. Furthermore, Suwazono et al. in a longitudinal study among Japanese workers have found 1.35 times increased risk of diabetes in the alternating shift work compared with the day-shift work [35].

Strength of the present study is that the subjects with prediabetes completed the PSQI questionnaire before they knew the results of the oral glucose tolerance test and the diagnosis of prediabetes had no effect on the participants' perceived quality of sleep. The limitations of the present study include its cross-sectional design and the number of studied subjects. Sleep quality has been assessed only subjectively and sleep disorders and obstructive sleep apnea have not been studied, as well.

## 5. Conclusion

In conclusion, this study provides evidences that subjects with poor sleep quality are more likely to develop prediabetes than people with good sleep quality. Implementation of sleep hygiene principles and regulation of sleep/work pattern can reduce the risk of prediabetes in susceptible population. Waking time was not a predictor of prediabetes in the present study. However, more longitudinal studies are necessary to understand the association of waking time and prediabetes.

## Figures and Tables

**Table 1:** PSQI scores of the study subjects.

	Normal	Prediabetes	*Z* value	*P* value
Subjective sleep quality	0.98 ± 0.64	1.00 ± 0.64	0.41	0.681
Sleep latency	1.15 ± 0.94	1.10 ± 0.91	0.65	0.514
Sleep duration	0.77 ± 0.78	0.89 ± 0.72	2.72	0.006
Habitual sleep efficiency	0.37 ± 0.77	0.33 ± 0.73	0.99	0.320
Sleep disturbances	1.09 ± 0.51	1.18 ± 0.51	2.34	0.019
Use of sleep medication	0.21 ± 0.62	0.26 ± 0.66	1.71	0.087
Daytime dysfunction	1.35 ± 0.83	1.30 ± 0.81	0.82	0.408

Data are presented as mean ± SD.

**Table 2:** Relationship between sleep patterns and prediabetes.

Variable	Total	Normal	Prediabetes	*χ* ^2^	df	*P* value
Sleep quality						
Good	52 (5.4)	44 (84.6)	8 (15.4)	3.757	1	0.053
Poor	904 (94.6)	654 (72.3)	250 (27.7)			
Bedtime						
At or before 12.00 am	788 (83.9)	580 (73.6)	208 (26.4)	0.280	1	0.597
After 12.00 am	151 (16.1)	108 (71.5)	43 (28.5)			
Waking time						
Before 6.00 am	113 (12.2)	78 (69.0)	35 (31.0)	12.161	2	0.002
Between 6 and 7 am	427 (46.2)	297 (69.6)	130 (30.4)			
After 7 am	384 (41.6)	306 (79.7)	78 (20.3)			
Sleep duration						
<6 hours	33 (3.5)	23 (69.7)	10 (30.3)	3.238	2	0.198
6–8 hours	668 (70.7)	478 (71.6)	190 (28.4)			
>8 hours	243 (25.8)	188 (77.4)	55 (22.6)			

Data are presented as number (percent).

**Table 3:** Logistic regression analysis of the relationship between “sleep” and “prediabetes.”

Variable	*β* value	SE	OR^*∗*^	95% CI	*P* value
Poor sleep quality	0.787	0.421	2.197	0.963–5.140	0.061
PSQI factors					
Subjective sleep quality	−0.006	0.127	0.994	0.776–1.274	0.965
Sleep latency	0.020	0.089	1.020	0.856–1.215	0.824
Sleep duration	0.059	0.105	1.061	0.863–1.305	0.573
Habitual sleep efficiency	−0.104	0.116	0.901	0.718–1.130	0.368
Sleep disturbances	0.122	0.158	1.130	0.829–1.542	0.440
Use of sleep medication	−0.018	0.123	0.982	0.772–1.249	0.883
Daytime dysfunction	−0.065	0.098	0.937	0.773–1.136	0.509
Late sleep onset	0.278	0.223	1.320	0.852–2.440	0.214
Waking time					
6-7 am			1		
<6 am	−0.245	0.257	0.783	0.473–1.297	0.342
>7 am	−0.265	0.187	0.767	0.531–1.107	0.157
Sleep duration					
6–8 hours			1		
<6 hours	−0.169	0.456	0.845	0.345–2.067	0.712
>8 hours	−0.238	0.197	0.788	0.536–1.159	0.226

^*∗*^Adjusted for age, gender, body mass index, and metabolic syndrome.
